# Safety and Tolerability of tDCS across Different Ages, Sexes, Diagnoses, and Amperages: A Randomized Double-Blind Controlled Study

**DOI:** 10.3390/jcm12134346

**Published:** 2023-06-28

**Authors:** Derrick M. Buchanan, Sarah Amare, Genevieve Gaumond, Amedeo D’Angiulli, Philippe Robaey

**Affiliations:** 1 Department of Neuroscience, Carleton University, Ottawa, ON K1S 5B6, Canada; matthewbuchanan@cmail.carleton.ca (D.M.B.);; 2 Neuroscience of Imagination Cognition Emotion Research Lab, Carleton University, Ottawa, ON K1S 5B6, Canada; 3 Neuropsychiatric Lab, Children’s Hospital of Eastern Ontario, Ottawa, ON K1H 8L1, Canada; 4 Department of Psychiatry, University of Ottawa, Ottawa, ON K1N 6N5, Canada

**Keywords:** adolescents, children, tdcs, safety, tolerability

## Abstract

Transcranial direct current stimulation (tDCS) is a non-invasive brain stimulation technique with substantial evidence for its safety and tolerability in adults. However, less than 5% of published tDCS research is in pediatrics. Our primary objective was to investigate tDCS safety, tolerability, and acceptability in a sample of children and adults. We hypothesized that children and adults would be equal with regard to tDCS safety, tolerability, and acceptability. We tested this hypothesis using a Bayesian approach. Sixty participants aged 6–45 (balanced for sex) participated in a randomized double-blind controlled trial. They were randomly assigned to two ten-minute tDCS sessions with varying amperages and electrode locations. The primary outcome measure of this study was the intensity of 13 potential side effects evaluated at six different time points spanning two weeks. Independent sample Bayes factor tests were conducted between children/adults, males/females, clinical/healthy, and low/high amperage groups. As predicted, there was moderate support for the null hypothesis in all between-group analyses. There were no serious adverse events or dropouts, and the number needed to treat for an additional harmful outcome was 23. This study provided evidence supporting the overall short-term safety, tolerability, and acceptability of tDCS including amperages up to 2 mA and different electrode placements.

## 1. Introduction

Transcranial direct current stimulation (tDCS) is a non-invasive brain stimulation technique with substantial evidence for its safety and tolerability in adults for up to 2 mA [1,2,3,4,5,6] and in some cases up to 4 mA [7]. Perhaps the most influential in regard to safety evidence is the early 2007 review from Poriesz et al. [2], and the more recent 2016 review from Bikson et al. [8]. Bikson’s review provided substantial evidence for safety in adults with over 7000 subjects and 33,000+ total sessions, however, it also revealed that less than 5% of all published tDCS literature is in pediatrics, and less than 2% of subjects were under the age of 18. This disproportionately low representation of children in the literature may be partially due to the scarcity of robust pediatric safety evidence compared to adults. Within this narrow 5% margin, only a handful of studies actually measured or assessed safety and tolerability in children [9,10,11,12,13,14,15,16,17,18]. That being said, there is certainly growing evidence for tDCS safety and tolerability in youth [19]. The most representative pediatric sample to date is also from Bikson et al. [8] reported on 2800 sessions in nearly 500 children: no serious adverse events were reported. Even more recently, in 2020, the largest single-site sample was published by Zewdie et al. [19] which consisted of 612 tDCS sessions in 92 children: no serious adverse events were reported. Finally, a 2021 systematic review on the safety and tolerability of tDCS in youth reported 12 studies, including a total of 1067 sessions in 156 children [20]. Although this review included fewer participants than Bikson et al. [8], it is uniquely important because the 12 studies that were reviewed rigorously evaluated safety using objective measures such as neuroimaging (i.e., magnetic resonance imaging (MRI), electroencephalography, electromyography, and transcranial magnetic stimulation), as well as critical medical evaluations such as vital signs (heart rate and blood pressure), or decline in cognitive/mental status based on neurological interview or cognitive tasks. The results of this systematic review seem to indicate on all accounts that tDCS appears to be both safe and tolerable in youth (aged 5–17) within the 0.5–2 mA range when applied for 10–20 min for up to 20 sessions. However, it is still not clear how tDCS safety/tolerability may differ between age groups, sexes, clinical vs. healthy control groups, or by amperage.

Since Bikson et al.’s 2016 study [8], there has been a clear growth in the number of tDCS studies being conducted in children and adolescents (registered clinical trials: 127/1530 in children = 8% as of February 2023 compared to the 32/1530 = 2% that existed in September 2016 at the time of Bikson’s publication), but even as of 2020 Zewdie et al. [19] are still calling for more robust safety and tolerability evidence for tDCS in youth. Although the field has benefited greatly from the above reviews, the studies they report on remain limited by their retrospective nature, lack of randomization, and lack of blinding procedures, which makes them subject to various biases. In addition, at any level of risk, stronger evidence is necessary to substantiate safety for vulnerable groups such as children.

In clinical research, the gold standard is the double-blind randomized controlled trial. To our knowledge, no article has specifically investigated the safety and tolerability of tDCS in youth using a randomized double-blind design or been able to compare safety and tolerability in adults under the same protocol. To firmly establish the safety and tolerability of tDCS in children, we set out to conduct such an investigation; aimed at directly comparing children to adults. We hypothesized that tDCS would be equally safe and tolerable in children as it is in adults. Similar to most psychiatric treatments, safety and tolerability are first established in adults before the treatment is used in children. Therefore, we strategically designed the present study to test the equality of tDCS safety and tolerability in youth and adults under the same protocol; such that tDCS is so well evidenced in adults that if this is found to be equal in children it may help to rapidly accelerate the translation of adult tDCS protocols for children.

Given this strategic design, we chose a statistical approach that could confirm the equality of safety and tolerability between these two groups. Traditional null hypothesis significance tests cannot assess the degree to which the data favor the null hypothesis compared to the alternative hypothesis, it can only confirm or reject the null. Accordingly, we adopted a Bayesian framework to quantify the degree of probability that the data are consistent with the null hypothesis (H0) compared to the alternative hypothesis (H1). We used the Bayes factor which is the ratio between the marginal likelihoods of the null model and the alternative model to estimate how likely the hypothesis is that children and adults experience the same level of side effects during tDCS sessions [21,22,23,24,25,26,27,28,29,30]. Thus, the use of the Bayesian approach allowed us to draw meaningful conclusions about the equality between children and adults for the safety and tolerability of tDCS. We also balanced the pediatric and adult groups for sex so we could make similar comparisons for possible sex differences.

The present investigation primarily aimed to prospectively examine the safety, tolerability, and acceptability of children and adults by measuring tDCS-related adverse events, side effects, and study dropouts, respectively [see the review from [8] for the operationalization of these variables]. This was performed in a randomized controlled fashion with side effects being evaluated before, during, and after tDCS sessions. We hypothesized that tDCS would be equally safe (adverse events), tolerable (side effects), and acceptable (dropouts) between children and adults. To date, the safety and tolerability of tDCS in children and adults have never been compared in the same research trial. Given that 95% of tDCS research has been in adults, this direct comparison of children and adults is an important incremental step to translating adult protocols for children and fostering hypothesis testing in youth. This study also serves to communicate that there does not appear to be any greater risk of side effects in children compared to adults. According to a recent acceptability study that interviewed parents of children who underwent tDCS [31], potential risks and robust safety/tolerability were of paramount concern. Therefore, given the rigorous and prospective design of this trial, we expect that this article will serve as an important reference for researchers and clinicians to cite for parents and children considering undergoing tDCS in a research trial or clinical setting. We also settle questions regarding risk comparisons related to sex, clinical status, and stimulation amperage which are also relevant to researchers, clinicians, parents, and anyone undergoing tDCS.

Previous studies have demonstrated that males and females have unique experiences in pain perception [32,33]. Similarly, clinical subjects such as those with attention deficit hyperactivity disorder (ADHD) have been shown to be at a greater risk for side effects due to aberrant pain perception [34,35]. Therefore, we extended our primary analysis to directly compare the safety and tolerability of tDCS between males and females, and between healthy subjects and subjects with a clinical diagnosis (i.e., ADHD, autism spectrum disorder: ASD, see Table 1), across both age groups. Our objective was to compare the likelihood estimates of the null model and the alternative model as a function of age, sex, and clinical status. From a clinical perspective, we wanted to provide tDCS practitioners with different points of comparison, so that they could inform their patients that there was no more risk between children and adults than between males and females, or between healthy controls and ADHD patients.

Third, and lastly, we also measured the effect of different levels of tDCS amperage on safety, tolerability, and acceptability. Recent research demonstrated that titrating tDCS amperage can differentially affect brain physiology [36,37,38,39], but within the amperages routinely used in clinics, there was no evidence regarding if amperage would differentially affect the subject’s side effect ratings. We hypothesized that tDCS amperage would not differentially affect side effect ratings, adverse events, or dropouts, among any of the above groups using a double-blind randomized controlled design. Again, we wanted to provide a Bayesian estimate of the likelihood of the null model and the alternative model so that clinicians could compare it with those obtained between the groups. Finally, as part of our analysis on amperage, we also provided the Number Needed to Treat for an Additional Harmful Outcome (NNTH) for tDCS. The NNTH is an indicator of how many people can be exposed to a treatment before someone experiences a side effect as compared to a placebo (0 mA sham condition) and is commonly reported in pharmacological treatment trials [40]. This number allows researchers, clinicians, and patients to make a meaningful and easy-to-understand comparison between the risks of using tDCS as a treatment versus a pharmaceutical alternative, for instance.

## 2. Materials and Methods

### 2.1. Participants

Sixty participants (Table 1) were recruited from the Children’s Hospital of Eastern Ontario (CHEO) Mental Health Patient Service Unit outpatient clinic (Mood and Anxiety, ADHD teams) as well from the community. Inclusion criteria included: ages 6–17 and 18–45 years old, and no previous experience with tDCS. Healthy controls had no history of neuropsychiatric conditions, while clinical participants had some neurodevelopmental condition (i.e., ADHD, ASD). Exclusion criteria included: history of epilepsy or seizure, adverse history of migraine/headaches, unstable medical condition or any condition that may increase the risk associated with transcranial stimulation, pregnancy, cardiac condition/recent cardiac surgery, neurological conditions, brain tumor, electronic implant, metal braces, metal plates in the head. Before committing to the experiment, all participants were screened to ensure that they met these criteria. This screening was conducted verbally, in person, or by phone by a member of the research team. This study was jointly approved by both the Children’s Hospital of Eastern Ontario and Carleton University’s Research Ethics Boards. All participants provided informed consent. Participants under the age of 18 provided assent in addition to the informed consent of the parent or guardian. Two assent forms were available for children ages 6–12 and 12–17 for grade-specific readability. A parent was present during the entirety of their child’s research visit. Each session took place in either the Neuropsychiatry Lab at CHEO or the Neuroscience of Imagination Cognition Emotion Research Lab at Carleton University.

### 2.2. Procedure

We used a randomized double-blind design. All participants underwent two successive 10 min tDCS sessions. In each session, the amplitude was randomly selected (0, 0.5, 1.0, and 2.0 mA). The researcher conducting the outcome measures and the participant (and parent) were blinded to the amperage. The currents were ramped up or down over the first and last 30 s of stimulation. We used six commonly used adult electrode montages which were randomized for each session. Our objective was not to test the effect of different montages, as there were no data to support such an effect. However, we wanted to use different montages to increase the clinical validity of the study, and thus the generalizability of its conclusions. The montages included: (1) anode over the motor cortex (C1) and cathode over the right frontopolar cortex above the eyebrow, (2) anode over the primary somatosensory cortex (P1) and cathode over the right frontopolar cortex above the eyebrow, (3) anode over the dorsolateral prefrontal cortex (DLPFC, F3) and cathode over the right frontopolar cortex above the eyebrow, (4) anode over the primary visual cortex (Oz) and cathode over (Cz) central midline, (5) anode over the temporal cortex (T3) and cathode over (T4) right temporal, (6) anode over the parietal cortex (P6–P8) and cathode over (Cz) central midline. Electrode locations given in brackets correspond to the standard electroencephalography 10–20 system [41] and were located using a flexible tape measure. Once again, the point of testing these six locations was not to measure differences between all of them, but rather to maximize the transferability and ecological validity of our study. In this way, testing only one montage would be a limitation to our objective.

### 2.3. Materials

Participants were seated upright in an office chair with the stimulation device placed on a desk behind them and out of site. We used the neuroConn DC stimulation device (Ilmenau, Germany). This device has a built-in safety feature that prevents it from running if the impedance is too high, and therefore electrode impedance was always kept below 5 Kohms. Each rubber electrode was covered in a 5 × 7 cm sponge soaked in a saline solution containing 0.9% NaCl. One or two large adjustable rubber bands were placed around the participant’s head to hold the electrodes in place. Between the two sessions, the participants were given a one-hour break during which they were allowed to sit quietly, watch videos, or play with toys or a videogame in the laboratory or an adjacent office.

### 2.4. Outcome Measures

The primary outcome measures used in this study were the intensity of 13 different side effects collected at six different time points: one week before the tDCS sessions (T1), immediately before the first tDCS session (T2), immediately after their first session (T3), after a one hour break just before the second session (T4), immediately after the second session (T5), and one week following the tDCS sessions (T6). The 13 side effects were quantitatively evaluated using a questionnaire adapted from Poreisz [2] and Brunoni [4]. The intensity of the side effects were rated on a Likert scale from 0–5 (0 nothing at all, 1 very mild, 2 mild, 3 moderate, 4 moderate to severe, 5 severe). These side effects included: feeling unwell, headaches, changes in concentration, being sad or wanting to cry, being anxious or nervous, visual disturbances, tiredness, scalp pain, scalp tingling, scalp itching, scalp burning, feeling nausea or needing to throw up, and trouble sleeping/feeling wakeful. The question ‘trouble sleeping/feeling wakeful’ was varied based on the time point such that children are not going to sleep at T3, T4, or T5; therefore, a question of wakefulness is more relevant; and at time points such as T1, T2, and T6 it was relevant if the child, with ADHD or otherwise, had difficulty sleeping. In addition to the 13-item questionnaire, two open-ended qualitative questions were offered to address any other side effects, concerns, or notes from the participant or their parent (i.e., being tired from the night before, or if their scalp was itchy from dandruff, as well as any major changes in routine such as one participant crying the week before tDCS because their pet died). Finally, a physical assessment of the scalp was conducted before and after each tDCS session. This included checking for redness, dryness, blistering, and burns, and recording if there is any pain at the site.

### 2.5. Statistical Analysis

#### 2.5.1. Primary Analysis on Age

We quantified the degree of probability that the data were consistent with the null hypothesis (H0) compared to the alternative hypothesis (H1) using independent sample Bayes tests for each of the 13 side effects and each of the six time points separately, with age as the between factor. In the statistical software we used (SPSS Version 29) Bayes factors >1 support the null hypothesis, and Bayes factors <1 support the alternative hypothesis. It is important to note this because Bayes factors are often reported the other way around. Our Bayes analysis was further supplemented by a multivariate analysis of variance (MANOVA) on children vs. adult side effect ratings across all six time points and all 13 side effects, and a series of univariate ANOVAs for each time point and side effect (see Supplementary Materials: Table S1). The analyses of variance were mainly confirmatory, providing partial effect sizes. It is also worth noting that, in our best attempt, to disprove our hypotheses that our various groups (child and adults, female and males, clinical and controls) share equal tolerance to tDCS, we did not perform multiple comparison corrections on our ANOVAs throughout this study. Given that we support the null hypothesis, not performing multiple comparison corrections is actually a more robust test against the null hypothesis.

#### 2.5.2. Comparison with Sex and Clinical Status

We performed the same analyses as in Section 2.5.1 for sex and clinical status, as for age group to provide a comparator.

#### 2.5.3. Secondary Analysis on Amperage

We again used independent sample Bayes tests to compare low versus high tDCS amperage. In this analysis, we pooled together the sham condition and the 0.5 mA conditions to create a low amperage group (*n* = 25, mean amplitude = 0.30 mA, standard deviation = 0.25 mA), and the one and two mA conditions to create a High Amperage group (*n* = 35, mean amplitude = 1.34 mA, standard deviation = 0.25 mA), based on each participant’s first tDCS session. Consistent with our earlier analyses, this analysis was supplemented by a multivariate analysis of variance (MANOVA) across all 13 side effects and two time points (T2: immediately before the first tDCS session, T3: immediately after the first session) as within factors, and with tDCS amperage (low and high amperage) as between factors. We also performed a series of repeated measure ANCOVA on amperage (low vs. high) and the 13 side effects measured at two time points (T2: immediately before the first tDCS session, T3: immediately after the first session) using the side effect ratings at T1 (one week before the session) as covariates.

#### 2.5.4. Number Needed to Treat for an Additional Harmful Outcome

As part of our analysis on amperage, we computed the Number Needed to Treat for an Additional Harmful Outcome (NNTH). NNTH is commonly reported in drug treatment trials [40] making it a meaningful metric for comparing the safety/lack of harm of tDCS related to common pharmacological interventions. NNTH is an indicator of how many people can be exposed to a treatment before someone experiences a side effect as compared to a placebo. NNTH was computed from (*n* = 10) participants in the placebo 0 mA condition and (*n* = 50) participants in the active 0.5–2 mA condition. Due to the possibility of carryover effects, NNTH was computed using data from the first tDCS session only. It was computed based on the side effects reported by low vs. high amperage groups immediately after their first tDCS session. The total number of all side effects rated above zero were divided by the total number of responses for each of the placebo and active conditions, respectively. Then the absolute difference between those two quotients was multiplied by 100 to provide the NNTH.

## 3. Results

### 3.1. Primary Analysis of tDCS Tolerability

#### 3.1.1. Children vs. Adults

We conducted independent sample Bayes tests for 13 side effects for each of the six time points as within factors, and with age as the between factor (Table 2). This yielded an average Bayes factor of 3.09 (standard deviation = 1.47) providing moderate support for the null hypothesis. A MANOVA comparing the pediatric group with the adult group across all six time points and 13 side effects showed no significant difference between the age groups across time: F (5, 290) = 1.719, *p* = 0.130, partial η^2^ = 0.029. See Table S1 for additional univariate ANOVA results.

**Figure 1:**
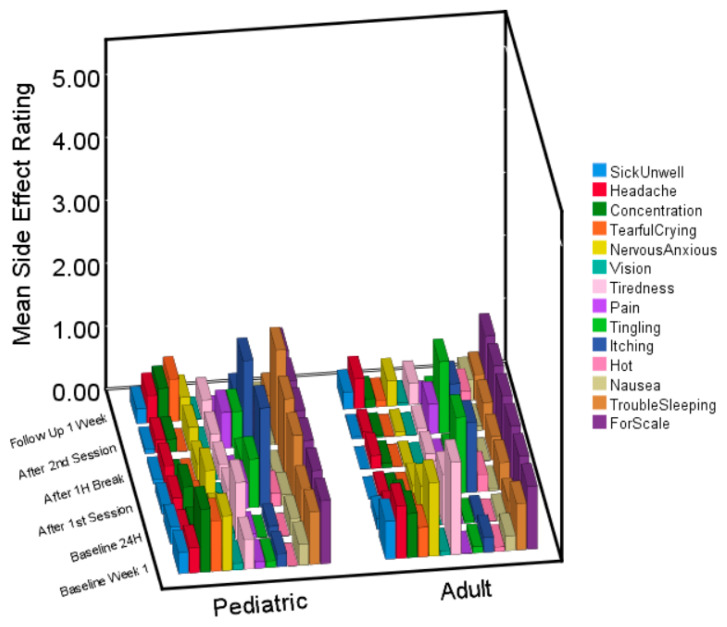
3D bar graph of side effects at each time point for children and adults. Average ratings for the pediatric and adult sample at each time point for the 13 different side effects. The 14th variable is a scale at a rating of 1, for comparison.

Moreover, as demonstrated visually in Figure 1, mean side effect ratings for both children and adults rarely exceeded a maximum rating of one ‘mild’ in each group. Therefore, even if there were some significant differences, it is the difference between a ‘mild’ and ‘very mild’ side effect rating. From a practical and clinical point of view, such a difference would have little clinical significance.

#### 3.1.2. Males vs. Females

As a comparison point, we conducted independent sample Bayes tests for 13 side effects for each of the six time points as within factors, and with males vs. females as between factors (Table 2). This yielded a corresponding average Bayes factor of 3.45 (standard deviation = 1.31), close to the estimates for the age effect. Then we conducted a MANOVA to compare the average side effect rating of males and females across all six time points and 13 side effects. In this case, we did observe a significant difference in side effects between males and females across time: F (5, 290) = 2.670, *p* = 0.022, partial η^2^ = 0.044. However, follow-up analyses showed that this effect of sex was only significant at Time 1 (one week before the tDCS session): F (1, 58) = 4.873, *p* = 0.031, partial η^2^ = 0.077. Therefore, this difference cannot be due to tDCS. See Table S1 for univariate ANOVA results and Figure S1 for a visualization of the data.

#### 3.1.3. Controls vs. Clinical

**Figure 2:**
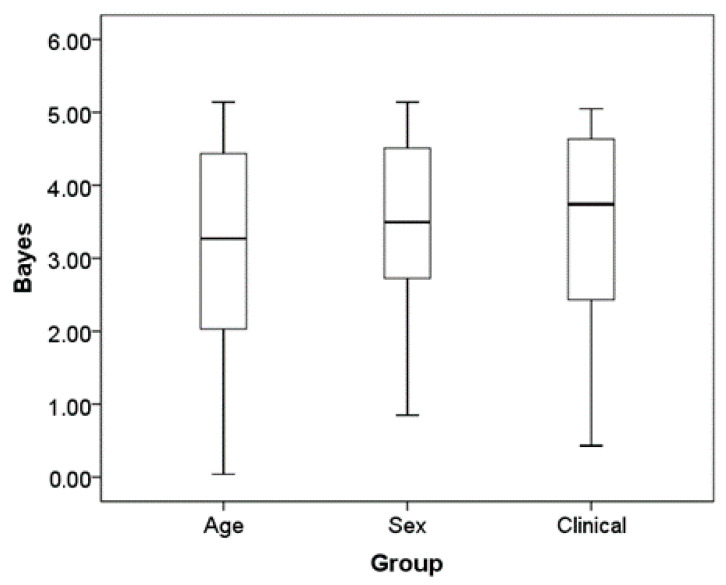
Boxplot of Bayesian factor average for age sex and clinical status. Boxplots of the distribution of the Bayes factor for each group difference in the ratings across the six times and 13 side effects.

Finally, we repeated the same analysis one more time, but this time comparing subjects with a clinical diagnosis vs. healthy subjects (Table 2). The average Bayes factor for this analysis was 3.39 (standard deviation = 1.40). For comparison, we have plotted the distribution of Bayes factors from each of our three Bayes analyses in Figure 2. The point of this visual comparison is to exemplify how the similarities between children and adults are about the same as between the other binary variables such as sex, and clinical status.

To provide converging evidence for our Bayes results, we conducted a MANOVA comparing the average side effect rating of clinically diagnosed subjects and healthy subjects across all six time points and 13 side effects. This did not reveal any differences between groups across time F (5, 290) = 0.858, *p* = 0.510, partial η^2^ = 0.015. Consistent with age, and sex, the overall side effect ratings also remained remarkably low. See Table S1 for univariate ANOVA results and Figure S2 for a visualization of the data.

### 3.2. Secondary Analysis of tDCS Tolerability Low vs. High Amperage

#### Side Effect Ratings for Low vs. High Amperage

We conducted independent sample Bayes tests for each of the 13 side effects and two time points (T3, T4) after the first tDCS session with tDCS amperage (low vs. high amperage groups) as between factors. This yielded an average Bayes factor of 3.41 (standard deviation = 1.21) providing moderate support for the null hypothesis that there is no difference in side effect ratings based on low vs. high tDCS amperage. This was further supported by a repeated measure MANCOVA using Amperage (low vs. high) as between factors and the 13 side effects at Time 3 and Time 4 as within factors repeated measures, and the side effect ratings at Time 1 as covariates. The results of this MANCOVA show that there was no significant effect of low vs. high amperage on side effect ratings from pre-to-post tDCS: F (1, 45) = 0.47, *p* = 0.495, partial η^2^ = 0.010.

Additionally, we were also interested in investigating whether or not high vs. low amperage would interact with any of the groups from our primary analysis (age, sex, clinical status). A repeated measure MANCOVA using Amperage (low vs. high) and Age (children vs. adult) as between factors and the 13 side effects at Time 3 and Time 4 as within factors repeated measures, and the side effect ratings at Time 1 as covariates, showed that the amperage by age interaction was not significant: F (1, 43) = 0.750, *p* = 0.391, partial η^2^ = 0.017. When age was replaced by sex in the previous analyses, the amperage by sex interaction was also not significant: F (1, 43) = 1.928, *p* = 0.172, partial η^2^ = 0.043. Finally, the amperage by clinical status interaction was also insignificant: F (1, 43) = 1.423, *p* = 0.239, partial η^2^ = 0.032.

### 3.3. Number Needed to Treat for an Additional Harmful Outcome

Finally, we computed the Number Needed to Treat for an Additional Harmful Outcome (NNTH) which equaled 23. This means that one in every 23 people who use tDCS may experience some side effect greater than a rating of zero when receiving a current of 0.5–2 mA, as compared to 0 mA (sham placebo).

### 3.4. Analysis of tDCS Safety and Acceptability

#### 3.4.1. Adverse Events and Dropouts

As a measurement of tDCS safety and acceptability, we assessed adverse events and study dropouts, respectively. There were no adverse events or dropouts from any subjects throughout the entire study. Accordingly, there were no differences between any groups, and the groups are exactly the same in this regard. In this sample, two sessions of tDCS were, therefore, completely safe and acceptable.

#### 3.4.2. Assessment of Skin and Scalp

Physical assessment of skin and scalp revealed that neither children nor adults experienced any dryness (0%), blistering (0%), or burns (0%) out of 120 total sessions. However, redness was seen under the electrodes in 8.3% of 60 sessions in children, and 18.3% of the 60 sessions in adults.

## 4. Discussion

This randomized double-blind trial provided robust and unbiased evidence for the short-term safety and tolerability of tDCS. Out of all the side effect ratings collected from 60 participants at different time points after tDCS (T3–T6), 84.2% were reported as zero. In addition, no participants dropped out or experienced any serious adverse events. Moreover, the overall average side effect rating was below one ‘very mild’ on a five-point Likert scale. Therefore, even if there were some significant differences, these differences bare no clinical significance.

Currently, a clear gap exists between the uptake of tDCS in adults and in children as 95% of the published tDCS research has been conducted in adults [8] and only 8% of the registered clinical trials are in pediatrics. Our experimental design allowed a direct comparison of safety, tolerability, and acceptability between children and adults, but also between males and females, and healthy vs. clinical participants. Moreover, our statistical design leveraged a Bayesian approach which allowed us to make direct comparisons between support for the null and alternative hypotheses; a more meaningful comparison than significance testing which alone cannot provide any evidence for the null hypothesis or equality between groups [26]. The Bayes analysis was also particularly useful for these comparisons as it is not subject to the statistical pitfalls of multiple comparisons. Based on all our analyses, short-term tDCS appears to be equally safe, tolerable, and acceptable among children and adults, males and females, and patients vs. controls. Additionally, based on research demonstrating how titrating tDCS amperage can differentially affect brain physiology [36,37,38,39] we also conducted analyses of tDCS amperage on side effect ratings; an outcome that has yet to be clearly reported in children in a controlled manner. tDCS amperage did not differentially affect the reported side effects as a function of age, sex, or clinical status. Given our knowledge of titration effects, this information is important such that practitioners may feel more confident about safely offering different levels of tDCS amperage to different cohorts.

One might object that we did not have enough power to detect very small effects, which is certainly true. Based on our data, a power analysis shows that in order to have an 80% chance of detecting an age effect size as small as 0.03 (average partial η) with an alpha of 5%, the projected sample size is close to 6000 subjects. Rather, we took a more strategic approach by demonstrating equality between youth and adults, a population where tDCS has extensively and repeatedly been proven to be safe and tolerable. Then, we used a more clinically meaningful metric for evaluating the safety of a treatment, the Number Needed to Treat for an Additional Harmful Outcome [42]. The NNTH for tDCS in our study was 23. This means that one out of every 23 patients a clinician treats with 0.5–2 mA tDCS may report some side effect (one out of the 13 side effects) to be greater than zero (on a scale of zero to five). These side effects would most likely be itchiness, tingling, and wakefulness/tiredness. Since our clinical sample was predominantly comprised of ADHD diagnoses it is meaningful to compare the NNTH for tDCS with the NNTH of frequently used ADHD medication. For example, methylphenidate, the most commonly used drug for ADHD, has an NNTH of four for appetite, seven for wakefulness/insomnia, nine for stomach aches, 10 for drowsiness, and 11 for dizziness in a systematic review of nearly 3000 children [43]. Therefore, based on this analysis, tDCS appears at least two to five times less harmful than the presently most commonly prescribed medication for ADHD.

The present study was limited to providing only two tDCS sessions per participant, whereas in clinical practice it is expected that upwards of 10–20 tDCS sessions will be required over the course of four weeks to achieve a lasting outcome [44,45]. To that end, it remains important for future treatment and efficacy trials to continue to monitor side effects longitudinally. That aside, the results of this trial clearly exemplify the lack of risk of acute pediatric tDCS justifying further uptake of tDCS into youth applications. This is especially useful for clinical trialists or cognitive neuroscientists aiming to answer pediatric-based research questions requiring brief exposures of tDCS. We believe that the present trial should greatly promote and facilitate the justification for such trials. In order to design truly efficacious tDCS treatment paradigms with strong effect sizes a significant amount of hypothesis testing must occur, and many hypotheses can be tested using only one or a few tDCS sessions [46,47,48]. Moreover, this is not just for tDCS use in pediatric ADHD, but also other cognitive or learning disorders such as dyslexia/dyscalculia, aphasias, or psychiatric disorders such as generalized anxiety disorder or major depressive disorder. One reason why hypothesis testing, even with a few tDCS sessions, is so critical is that a number of sham-controlled tDCS trials have failed to find significant differences between active and sham conditions; and those that have often reported weak effect sizes. Similar to what has been discovered using more researched non-invasive brain stimulation techniques such as repetitive transcranial magnetic stimulation (rTMS), a failed trial does not always mean that a treatment will never be effective. We postulate, as many others, that tDCS as a treatment will achieve stronger effect sizes once we identify the exact optimal parameters for a given disorder and person (i.e., dose optimization via individualized electrode placement and amperage to maximize e-field at target site [49,50], accurate MRI guided targeting [51,52], or even functional MRI targeting such as what emerged in recent rTMS research [53,54]). In the future, it is also likely that measuring electrophysiological responses to even a small number of tDCS sessions could predict treatment response. All of these hypotheses could be tested using a small number of tDCS sessions.

Overall, since the side effects of tDCS are so mild, and the likelihood of experiencing them is very low, tDCS should be considered more actively as an adjunct or alternative treatment option to medication and as a research tool. Our findings are in line with the results from our recent qualitative study that showed how tDCS is raising hope for parents of children with ADHD dealing with pharmacological treatment: These parents made it clear that understanding the safety, tolerability, and side effects of tDCS was imperative for accepting the treatment, provided that it is effective, relatively inexpensive, practical, and accessible [9,31]. Thankfully, the call for tDCS as a treatment option for ADHD may soon be answered as promising results from a number of clinical trials are starting to be published in adults [55] and in children [48,56,57].

## 5. Conclusions

The present study provided strong evidence supporting the overall short-term safety and tolerability of tDCS, including multiple electrode placements and direct current up to 2 mA, by comparing children vs. adults, males vs. females, and healthy vs. clinical participants. The results of the present study should help to facilitate and justify the clinical uptake and transferability of adult tDCS evidence to youth applications, as well as the development of child-specific hypothesis testing.

## Figures and Tables

**Table 1:** Participant demographics and electrode montage.

	Participant	Age	Sex	Population	Session 1 Location	Session 1 Amp	Session 2 Location	Session 2 Amp
Child	1	17	Male	Healthy	(P6–P8)	2	(Oz)	0
	2	14	Female	Healthy	(F3)	1	(P1)	1
	3	8	Male	Clinical, ADHD	(F3)	1	(P1)	2
	4	6	Female	Healthy	(Oz)	0.5	(F3)	2
	5	17	Male	Clinical, Asperger’s, Anxiety, Tourette	(C1)	0.5	(T3)	1
	6	15	Male	Healthy	(F3)	1	(F3)	2
	7	11	Male	Clinical, ADHD, ADD, PTSD, ODD, GAD, Depression	(P1)	1	(T3)	1
	8	16	Female	Clinical, Bipolar type 2, ADHD, ASD	(C1)	0.5	(T3)	1
	9	16	Male	Clinical, ADHD, ODD, GAD	(C1)	1	(T3)	0.5
	10	17	Female	Clinical ADHD	(F3)	2	(P6–P8)	0
	11	11	Male	Clinical, ADHD, OCD, LD	(F3)	1	(F3)	0.5
	12	6	Male	Healthy	(F3)	2	(T3)	2
	13	8	Male	Healthy/Gifted	(T3)	1	(P1)	2
	14	6	Female	Healthy	(C1)	0.5	(P1)	2
	15	8	Female	Healthy	(P1)	0.5	(P6–P8)	1
	16	12	Male	Clinical, ADHD	(Oz)	1	(T3)	2
	17	8	Male	Clinical, ADHD	(F3)	0	(T3)	2
	18	13	Female	Healthy	(F3)	0	(P6–P8)	2
	19	11	Female	Clinical, ADHD, ODD, GAD	(C1)	0	(T3)	2
	20	6	Male	Healthy	(F3)	2	(T3)	2
	21	10	Female	Clinical, ADHD	(T3)	2	(P1)	0
	22	7	Female	Healthy	(Oz)	1	(P1)	0
	23	14	Male	Healthy	(T3)	1	(Oz)	2
	24	13	Female	Healthy	(F3)	0.5	(C1)	2
	25	11	Female	Healthy	(C1)	0	(T3)	2
	26	8	Male	Clinical, ADHD, ASD	(F3)	1	(T3)	0
	27	10	Female	Healthy	(P6–P8)	1	(T3)	0
	28	13	Female	Healthy	(F3)	0.5	(P6–P8)	1
	29	10	Male	Healthy	(F3)	0.5	(T3)	1
	30	13	Female	ADHD Anxiety	(P1)	1	(P1)	0
	Average/Count	11.17	15 Male	14 Clinical	13 DLPFC, 6 MC, 2 PC, 3 SC, 3 TC, 3 VC	0.9	3 DLPFC, 1 MC, 4 PC, 7 SC, 13 TC, 2 VC	1.2
Adult	1	18	Male	Clinical, Dyslexia	(C1)	0	(P1)	0.5
	2	24	Male	Healthy	(Oz)	0	(Oz)	1
	3	26	Female	Healthy	(P6–P8)	0	(T3)	1
	4	18	Female	Clinical, CAPD	(Oz)	0.5	(C1)	1
	5	21	Female	Clinical, ADHD	(P6–P8)	1	(Oz)	2
	6	19	Female	Healthy	(P1)	1	(Oz)	1
	7	19	Female	Healthy	(P1)	2	(C1)	1
	8	19	Male	Clinical, ADHD, GAD	(T3)	2	(P6–P8)	0.5
	9	18	Female	Clinical, ADHD, RSPD, GAD, PDD NOS	(P1)	0.5	(Oz)	0
	10	45	Female	Healthy	(T3)	1	(P1)	1
	11	41	Female	Healthy	(C1)	1	(F3)	2
	12	21	Male	Healthy	(F3)	2	(P6–P8)	0.5
	13	28	Male	Healthy	(Oz)	0	(T3)	1
	14	21	Male	Healthy	(F3)	1	(T3)	2
	15	21	Male	Clinical, ADHD	(P6–P8)	0.5	(P6–P8)	1
	16	24	Male	Healthy	(P6–P8)	0.5	(P6–P8)	2
	17	23	Male	Healthy	(C1)	1	(P1)	2
	18	20	Female	Healthy	(P1)	2	(Oz)	2
	19	26	Male	Clinical, MDD, ADHD, GAD	(P6–P8)	1	(F3)	2
	20	21	Male	ADD	(C1)	2	(F3)	2
	21	25	Female	Healthy	(C1)	1	(T3)	2
	22	25	Male	Healthy	(Oz)	0	(Oz)	2
	23	24	Female	Healthy	(C1)	0.5	(P6–P8)	0
	24	24	Female	Healthy	(C1)	1	(T3)	2
	25	25	Female	Clinical, ADHD	(P1)	1	(F3)	0
	26	24	Female	Healthy	(C1)	0	(F3)	0
	27	23	Male	Healthy	(P1)	2	(Oz)	2
	28	23	Male	Healthy	(F3)	2	(F3)	0
	29	32	Male	Healthy	(T3)	0.5	(C1)	1
	30	27	Female	Clinical, ADHD	(F3)	0.5	(T3)	2
	Average/Count	24.17	15 Male	10 Clinical	4 DLPFC, 8 MC, 5 PC, 6 SC, 3 TC, 4 VC	0.92	6 DLPFC, 3 MC, 5 PC, 3 SC, 6 TC, 7 VC	1.22

Oz = visual cortex, F3 = dorsolateral prefrontal cortex, T3 = temporal cortex, P6–P8 = parietal cortex, C1 = motor cortex, P1 = somatosensory cortex, ADHD = attention deficit hyperactivity disorder, ASD = autism spectrum disorder, ODD = oppositional defiance disorder, GAD = generalized anxiety disorder, MDD = major depression disorder, PTSD = post-traumatic stress disorder, CAPD = central auditory processing disorder.

**Table 2:** Bayes factor of each group effect.

		Age	Sex	Clinical Status
Side Effect	Time	Bayes	Bayes	Bayes
Sick/Unwell	T1	3.000	3.803	3.534
	T2	4.470	5.065	3.017
	T3	2.460	4.288	3.483
	T4	2.030	5.144	4.863
	T5	4.400	4.404	4.923
	T6	5.080	2.858	5.051
Headache	T1	1.245	2.635	4.943
	T2	4.773	4.773	4.516
	T3	2.730	1.462	3.867
	T4	4.760	4.760	4.907
	T5	3.839	3.839	2.301
	T6	5.110	4.337	4.850
Difficulty Concentrating	T1	3.659	4.189	0.432
	T2	3.533	4.936	1.134
	T3	0.609	5.058	2.900
	T4	2.027	2.027	4.863
	T5	2.043	2.043	5.021
	T6	1.585	4.527	4.423
Tearful/Crying	T1	3.098	4.291	3.110
	T2	5.144	5.144	3.630
	T3	3.224	1.372	4.077
	T4	.	.	.
	T5	.	.	.
	T6	0.558	2.819	1.772
Nervous/Anxious	T1	3.575	4.131	4.871
	T2	3.680	4.839	5.029
	T3	0.929	0.929	4.126
	T4	1.462	4.104	4.968
	T5	1.396	1.396	3.680
	T6	5.109	4.329	1.895
Vision	T1	4.967	2.105	4.365
	T2	3.270	3.270	2.558
	T3	2.027	5.144	1.238
	T4	.	.	.
	T5	.	.	.
	T6	3.270	3.270	2.558
Tiredness	T1	0.044	1.029	4.735
	T2	4.947	1.016	1.741
	T3	3.860	3.860	0.523
	T4	4.517	3.043	0.662
	T5	3.626	4.716	3.513
	T6	5.092	3.974	5.017
Pain	T1	3.270	3.270	2.558
	T2	3.270	3.270	2.558
	T3	1.049	3.185	1.382
	T4	3.270	3.270	3.742
	T5	4.809	4.264	2.747
	T6	.	.	.
Tingling	T1	5.144	1.223	1.964
	T2	3.270	3.270	3.742
	T3	1.338	5.105	5.025
	T4	3.406	1.316	1.305
	T5	1.022	4.493	4.769
	T6	2.027	5.144	2.732
Itching	T1	4.961	3.705	4.064
	T2	3.472	3.472	4.501
	T3	2.433	5.068	1.376
	T4	4.219	2.302	4.428
	T5	1.207	0.848	1.844
	T6	2.169	3.518	4.167
Hot	T1	3.270	3.270	3.742
	T2	.	.	.
	T3	4.874	3.150	4.525
	T4	3.270	3.270	2.558
	T5	0.684	4.640	4.174
	T6	3.270	3.270	2.558
Nausea	T1	4.701	3.137	4.415
	T2	2.498	4.753	5.051
	T3	2.746	5.144	4.767
	T4	3.270	3.270	3.742
	T5	4.404	4.404	1.964
	T6	3.981	3.981	4.262
Trouble Sleeping/Wakefulness	T1	4.940	0.957	4.991
	T2	1.756	3.092	1.596
	T3	1.252	1.649	1.309
	T4	0.780	1.381	4.464
	T5	0.214	2.417	0.450
	T6	4.823	5.108	3.426

“.” Indicates that all the ratings were zero.

## Data Availability

All relevant data related to this study are reported in the manuscript or Supplementary Materials.

## Supplementary Materials

The following supporting information can be downloaded at: https://www.mdpi.com/article/10.3390/jcm12134346/s1, Figure S1: 3D bar graph of side effects at each time point for females and males.; Figure S2: 3D bar graph of side effects at each time point for clinical and healthy subjects.; Table S1: F statistics of each group effect and associated partial η, as well as Bayes factor; Table S2: Effect of Low vs. High amperage across the whole sample, and interactions with age, sex, and clinical status.
