# Supplementary material for: Safety and Tolerability of tDCS across Different Ages, Sexes, Diagnoses, and Amperages: A Randomized Double-Blind Controlled Study
Source: J Clin Med. 2023 Jun 28;12(13):4346. doi: 10.3390/jcm12134346 (PMC10342754; doi:10.3390/jcm12134346)
Supplement: Supplementary file 1 [file jcm-12-04346-s001.zip › jcm-2284472-supplementary.pdf]

Table S1. F statistics of each group effect and associated partial η, as well as Bayes factor.

		Age			Sex			Clinical Status		
Side effect	Time	$F_{1, 58}$	$\eta_{partial}$	Bayes	$F_{1, 58}$	$\eta_{partial}$	Bayes	$F_{1, 58}$	$\eta_{partial}$	Bayes
Sick/Unwell	T1	1.193	0.020	3.000	0.665	0.011	3.803	0.790	0.013	3.534
	T2	0.310	0.005	4.470	0.034	0.001	5.065	1.143	0.019	3.017
	T3	1.634	0.027	2.460	0.400	0.007	4.288	0.823	0.014	3.483
	T4	2.071	0.034	2.030	0.000	0.000	5.144	0.083	0.001	4.863
	T5	0.341	0.006	4.400	0.341	0.006	4.404	0.057	0.001	4.923
	T6	0.026	0.000	5.080	1.300	0.022	2.858	0.000	0.000	5.051
Headache	T1	3.180	0.052	1.245	1.482	0.025	2.635	0.048	0.001	4.943
	T2	0.164	0.003	4.773	0.164	0.003	4.773	0.247	0.004	4.516
	T3	1.402	0.024	2.730	2.814	0.046	1.462	0.590	0.010	3.867
	T4	0.171	0.003	4.760	0.171	0.003	4.760	0.064	0.001	4.907
	T5	0.644	0.011	3.839	0.644	0.011	3.839	1.751	0.029	2.301
	T6	0.015	0.000	5.110	0.375	0.006	4.337	0.090	0.002	4.850
Difficulty Concentrating	T1	0.751	0.013	3.659	0.452	0.008	4.189	**5.628**	0.088	0.432
	T2	0.829	0.014	3.533	0.091	0.002	4.936	3.365	0.055	1.134
	T3	**4.840**	0.077	0.609	0.037	0.001	5.058	1.232	0.021	2.900
	T4	2.071	0.034	2.027	2.071	0.034	2.027	0.083	0.001	4.863
	T5	2.054	0.034	2.043	2.054	0.034	2.043	0.013	0.000	5.021
	T6	2.630	0.043	1.585	0.281	0.005	4.527	0.293	0.005	4.423
Tearful/Crying	T1	1.121	0.019	3.098	0.398	0.007	4.291	1.075	0.018	3.110
	T2	0.000	0.000	5.144	0.000	0.000	5.144	0.730	0.012	3.630
	T3	1.032	0.017	3.224	2.959	0.049	1.372	0.473	0.008	4.077
	T4	.	.	.	.	.	.	.	.	.
	T5	.	.	.	.	.	.	.	.	.
	T6	**5.046**	0.080	0.558	1.331	0.022	2.819	2.342	0.039	1.772
Nervous/Anxious	T1	0.802	0.014	3.575	0.483	0.008	4.131	0.080	0.001	4.871
	T2	0.738	0.013	3.680	0.134	0.002	4.839	0.010	0.000	5.029
	T3	3.856	0.062	0.929	3.856	0.062	0.929	0.446	0.008	4.126
	T4	2.814	0.046	1.462	0.497	0.008	4.104	0.037	0.001	4.968
	T5	2.918	0.048	1.396	2.918	0.048	1.396	0.700	0.012	3.680
	T6	0.015	0.000	5.109	0.379	0.006	4.329	2.191	0.036	1.895
Vision	T1	0.077	0.001	4.967	1.986	0.033	2.105	0.322	0.006	4.365
	T2	1.000	0.017	3.270	1.000	0.017	3.270	1.513	0.025	2.558
	T3	2.071	0.034	2.027	0.000	0.000	5.144	3.164	0.052	1.238
	T4	.	.	.	.	.	.	.	.	.
	T5	.	.	.	.	.	.	.	.	.
	T6	1.000	0.017	3.270	1.000	0.017	3.270	1.513	0.025	2.558
Tiredness	T1	**11.308**	0.163	0.044	3.619	0.059	1.029	0.142	0.002	4.735
	T2	0.086	0.001	4.947	3.649	0.059	1.016	2.383	0.039	1.741
	T3	0.632	0.011	3.860	0.632	0.011	3.860	**5.172**	0.082	0.523
	T4	0.286	0.005	4.517	1.160	0.020	3.043	**4.617**	0.074	0.662
	T5	0.771	0.013	3.626	0.191	0.003	4.716	0.803	0.014	3.513
	T6	0.022	0.000	5.092	0.568	0.010	3.974	0.015	0.000	5.017
Pain	T1	1.000	0.017	3.270	1.000	0.017	3.270	1.513	0.025	2.558
	T2	1.000	0.017	3.270	1.000	0.017	3.270	1.513	0.025	2.558
	T3	3.575	0.058	1.049	1.058	0.018	3.185	2.911	0.048	1.382
	T4	1.000	0.017	3.270	1.000	0.017	3.270	0.663	0.011	3.742
	T5	0.148	0.003	4.809	0.413	0.007	4.264	1.353	0.023	2.747
	T6	.	.	.	.	.	.	.	.	.

Tingling	T1	0.000	0.000	5.144	3.222	0.053	1.223	2.109	0.035	1.964
	T2	1.000	0.017	3.270	1.000	0.017	3.270	0.663	0.011	3.742
	T3	3.016	0.049	1.338	0.017	0.000	5.105	0.011	0.000	5.025
	T4	0.910	0.015	3.406	3.055	0.050	1.316	3.041	0.050	1.305
	T5	3.636	0.059	1.022	0.297	0.005	4.493	0.127	0.002	4.769
	T6	2.071	0.034	2.027	0.000	0.000	5.144	1.365	0.023	2.732
Itching	T1	0.079	0.001	4.961	0.723	0.012	3.705	0.480	0.008	4.064
	T2	0.867	0.015	3.472	0.867	0.015	3.472	0.254	0.004	4.501
	T3	1.661	0.028	2.433	0.033	0.001	5.068	2.921	0.048	1.376
	T4	0.436	0.007	4.219	1.785	0.030	2.302	0.290	0.005	4.428
	T5	3.251	0.053	1.207	**4.067**	0.066	0.848	2.252	0.037	1.844
	T6	1.919	0.032	2.169	0.838	0.014	3.518	0.424	0.007	4.167
Hot	T1	1.000	0.017	3.270	1.000	0.017	3.270	0.663	0.011	3.742
	T2	.	.			.			.	
	T3	0.118	0.002	4.874	1.083	0.018	3.150	0.242	0.004	4.525
	T4	1.000	0.017	3.270	1.000	0.017	3.270	1.513	0.025	2.558
	T5	**4.568**	0.073	0.684	0.226	0.004	4.640	0.421	0.007	4.174
	T6	1.000	0.017	3.270	1.000	0.017	3.270	1.513	0.025	2.558
Nausea	T1	0.198	0.003	4.701	1.092	0.018	3.137	0.297	0.005	4.415
	T2	1.601	0.027	2.498	0.174	0.003	4.753	0.000	0.000	5.051
	T3	1.389	0.023	2.746	0.000	0.000	5.144	0.127	0.002	4.767
	T4	1.000	0.017	3.270	1.000	0.017	3.270	0.663	0.011	3.742
	T5	0.341	0.006	4.404	0.341	0.006	4.404	2.109	0.035	1.964
	T6	0.564	0.010	3.981	0.564	0.010	3.981	0.375	0.006	4.262
Trouble	T1	0.089	0.002	4.940	3.786	0.061	0.957	0.026	0.000	4.991
Sleeping/	T2	2.397	0.040	1.756	1.124	0.019	3.092	2.582	0.043	1.596
Wakefulness	T3	3.168	0.052	1.252	2.539	0.042	1.649	3.034	0.050	1.309
	T4	**4.262**	0.068	0.780	2.943	0.048	1.381	0.272	0.005	4.464
	T5	**7.340**	0.112	0.214	1.676	0.028	2.417	**5.530**	0.087	0.450
	T6	0.141	0.002	4.823	0.016	0.000	5.108	0.859	0.015	3.426

Significant effects are in bold; . indicates that all the ratings were zero.

Table S1 presents the 234 (13X6X3) comparisons between 3 groups (children vs adults, males vs females, ADHD vs healthy controls) of 13 side effects for each of the six time points. A total of 11 significant differences were present across all 234 comparisons (.047%), which is less than expected even by chance (i.e., alpha .05). These differences also did not appear to cluster on any specific side effect or time point but rather appeared quite sporadically. No group difference survived a multiple comparison correction.

Table S2: Effect of Low vs. High amperage across the whole sample, and interactions with age, sex and clinical status

		Amperage			Age X Amp		Sex X Amp		Clinical Status X Amp	
Side effect	Time	$F_{1,57}$	$\eta_{partial}$	Bayes	$F_{1,55}$	$\eta_{partial}$	$F_{1,55}$	$\eta_{partial}$	$F_{1,55}$	$\eta_{partial}$
Sick Unwell	T2	0.057	0.001	4.322	0.056	0.001	3.695	0.063	3.191	0.055
	T3	1.167	0.020	3.012	0.970	0.017	0.146	0.003	0.586	0.011
	T4	1.733	0.030	2.626	0.594	0.011	0.017	0.000	0.072	0.001
Headache	T2	2.280	0.038	1.439	1.660	0.029	0.238	0.004	0.271	0.005
	T3	0.339	0.006	4.325	0.151	0.003	0.418	0.008	0.419	0.008
	T4	0.558	0.010	3.485	2.917	0.050	0.004	0.000	0.100	0.002
Concentrating	T2	0.056	0.001	5.026	0.030	0.001	0.304	0.006	0.002	0.000
	T3	0.901	0.016	3.380	1.610	0.028	0.002	0.000	0.001	0.000
	T4	0.062	0.001	4.949	0.003	0.000	0.203	0.004	1.370	0.024
Tearful	T2	0.027	0.000	3.461	1.154	0.021	0.003	0.000	0.076	0.001
Crying	T3	3.575	0.059	0.784	1.383	0.025	3.156	0.054	0.733	0.013
	T4	.	.	.	.	.	.	.	.	.
Nervous	T2	0.371	0.006	1.287	3.937	0.067	0.071	0.001	0.408	0.007
Anxious	T3	0.236	0.004	2.903	0.107	0.002	0.396	0.007	0.032	0.001
	T4	0.049	0.001	4.795	0.472	0.009	1.383	0.025	0.374	0.007
Vision	T2	0.057	0.001	3.682	1.351	0.024	0.045	0.001	1.287	0.023
	T3	0.040	0.001	4.949	0.079	0.001	1.510	0.027	0.074	0.001
	T4	.	.	.	.	.	.	.	.	.
Tiredness	T2	1.701	0.029	3.093	1.736	0.031	2.543	0.044	0.203	0.004
	T3	0.008	0.000	5.070	1.042	0.019	0.198	0.004	0.152	0.003
	T4	1.131	0.019	2.964	1.965	0.034	0.037	0.001	1.890	0.033
Pain	T2	.	.	3.682	0.000	0.000	.	.	0.000	0.000
	T3	0.315	0.006	4.496	0.085	0.002	0.004	0.000	0.295	0.005
	T4	1.369	0.023	2.691	1.269	0.023	0.916	0.016	0.899	0.016
Tingling	T2	0.806	0.014	3.682	2.960	0.051	0.692	0.012	0.612	0.011
	T3	0.809	0.014	3.366	0.502	0.009	0.839	0.015	1.683	0.030
	T4	0.724	0.013	3.421	0.109	0.002	0.008	0.000	0.450	0.008
Itching	T2	**5.470**	0.088	2.619	0.148	0.003	1.780	0.031	1.414	0.025
	T3	2.228	0.038	2.712	0.792	0.014	0.000	0.000	0.773	0.014
	T4	2.034	0.034	1.147	0.234	0.004	1.243	0.022	0.076	0.001
Hot	T2	.	.	.	.	.	.	.	.	.
	T3	0.011	0.000	5.072	0.142	0.003	0.654	0.012	**4.689**	0.079
	T4	1.470	0.025	2.691	1.483	0.026	0.889	0.016	2.104	0.037
Nausea	T2	0.395	0.007	4.548	0.569	0.010	0.930	0.017	0.571	0.010
	T3	0.104	0.002	4.882	0.571	0.010	0.994	0.018	1.370	0.024
	T4	1.093	0.019	3.682	0.002	0.000	0.466	0.008	1.341	0.024
	T2	0.689	0.012	4.085	0.263	0.005	1.636	0.029	0.010	0.000
	T3	1.625	0.028	2.438	1.408	0.025	0.148	0.003	0.135	0.002

Trouble Sleeping/ Wakefulness	T4	1.984	0.034	2.078	0.157	0.003	0.006	0.000	0.159	0.003

Table S2: Significant effects are in bold; . indicates that all the ratings were zero.

Figure S1. 3D bar graph of side effects at each time point for females and males.

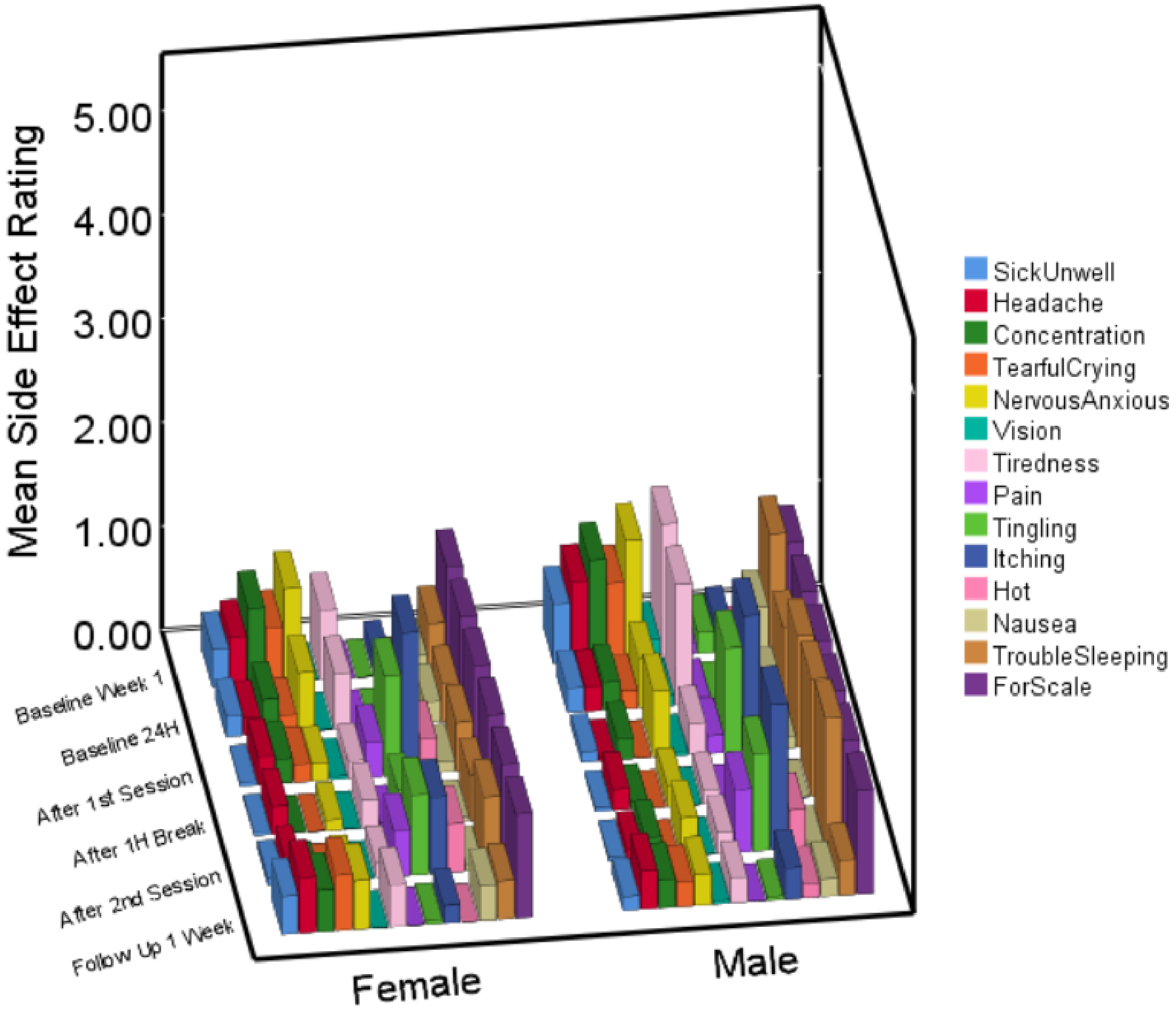

Figure S1: Average ratings for the female and male sample at each time point for the 13 different side effects. The 14th variable is a scale at a rating of 1, for comparison.

Figure S1 clearly illustrates that mean side effect ratings of both males and females rarely exceeded a maximum rating of 1 ‘mild’.

Figure S2. 3D bar graph of side effects at each time point for clinical and healthy subjects.

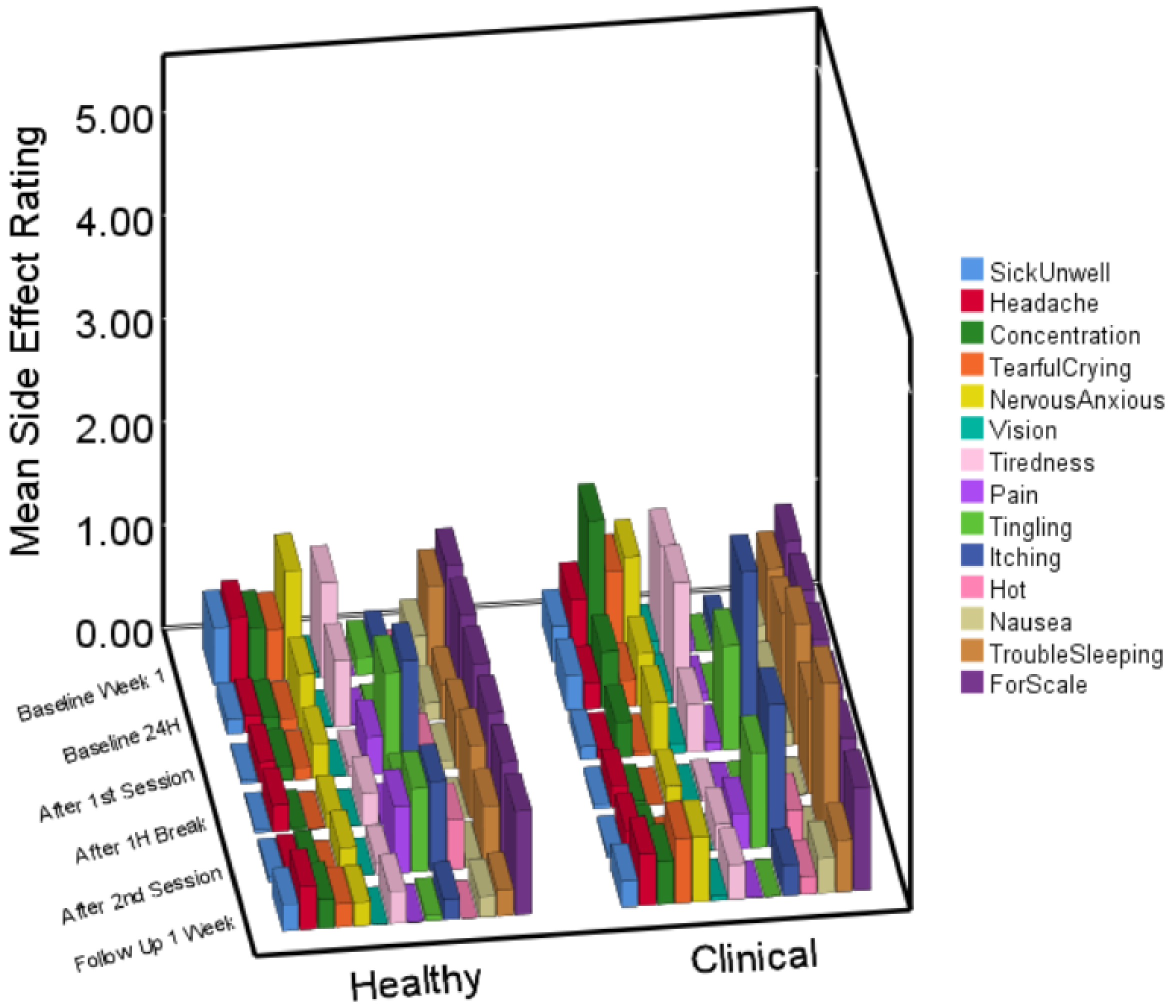

Figure S2: Average ratings for the control and clinical sample at each time point for the 13 different side effects. The 14th variable is a scale at a rating of 1, for comparison.

Figure S2 clearly illustrates that mean side effect ratings of both males and females rarely exceeded a maximum rating of 1 'mild'.
